# The Value of Health Economics and Outcomes Research in Prosthetics and Orthotics

**DOI:** 10.33137/cpoj.v4i2.35959

**Published:** 2021-09-21

**Authors:** TA Miller, S Wurdeman, R Paul, M Forthofer

**Affiliations:** 1 Department of Clinical and Scientific Affairs, Hanger Clinic, Austin, Texas, USA.; 2 College of Health and Human Services, University of North Carolina at Charlotte, Charlotte, North Carolina, USA.; 3 Department of Biomechanics, The University of Nebraska at Omaha, Omaha, Nebraska, USA.; 4 Department of Public Health Sciences, School of Data Science, University of North Carolina Charlotte, North Carolina, USA.

**Keywords:** Health Economics, Prosthetics, Orthotics, Outcomes Research, Rehabilitation

## Abstract

The demand has increased for evidence regarding the effectiveness and value of prosthetic and orthotic rehabilitation interventions. Clinicians and managers are under pressure to provide treatment recommendations and demonstrate effectiveness through outcomes. It is often assumed that rehabilitation interventions, including the provision of custom-made and custom-fit orthotic and prosthetic devices, are beneficial to patients. Assessing the value of orthotic and prosthetic services has become more critical to continue to ensure equitable access to needed services. Health economics and outcomes research methods serve as tools to gauge the value of prosthetic and orthotic rehabilitation interventions. The purpose of this article is to provide an overview of the current need of health economics and outcomes research in orthotics and prosthetics, to introduce common economic methods that assist to generate real-world evidence, and to discusses the potential value of economic methods for clinicians and clinical practice.

## INTRODUCTION

Rehabilitation is can be defined as a problem-solving process or service aimed at reducing disability or impairment experienced by an individual as a result of disability or injury ultimately to improve function.^1,2^ Unmet needs for rehabilitation services and health systems are often undervalued services**.2,3** Individuals who have a lower limb amputation (LLA) or require orthotic bracing experience numerous, overlapping difficulties with respect to overall physical health including functional recovery as well as social and mental health.^4–7^ As healthcare costs have increased, the economic burden associated with care for those with chronic conditions, especially functional impairment and disability, remains high.**8-11** Rehabilitation is essential if individuals are to regain functional independence, return to ADLs and good overall health whether it be in a post-amputation condition or a progressive neuromusculoskeletal condition. Yet, real-world evidence (RWE) on rehabilitation outcomes among those with LLA and neuromusculoskeletal conditions requiring orthotic intervention is sparse. There is a paucity of information relating to timing of orthosis or prosthesis receipt in the care pathway, effectiveness of interventions, overall costs and utilization. Hence, there is a need for more RWE on factors that influence outcomes to help inform clinical practice and guide clinicians, strengthen policy, and influence patient-health while being cost-effective.12,13 The purpose of this paper is to explicate health economics and outcomes research (HEOR) as a field, discuss recent applications in orthotics and prosthetics (O&P), and the need for continued health economic research.

Recent publications on economic science provided perspectives from consumers, providers, and manufacturers,^14–16^ which highlighted that health economic analyses and science is not about reducing access to essential O&P care but to optimize outcomes and access. Healthcare decision makers today are often faced with the need to select from multiple treatment options, the timing of any such treatment, or determine alternative appropriate care plans. However, the benefits and associated costs of these different interventions or plans can vary greatly. The benefits can be clinical, economic, or may include more humanistic outcomes. Humanistic outcomes, such as patient experiences, are more challenging to measure as they cannot be evaluated by clinicians but rather are patient-reported (e.g. pain and quality of life). HEOR applications (i.e. types of economic evaluations) include broad, scientifically vigorous methods and tools used to assess the effectiveness and impact of specific interventions (e.g. specific knee selection) in order to adequately compare and choose treatments or devices among the available options (Table 1).

**Table 1:** Examples of the basic types of economic evaluations and preference-based analyses with applications in O&P or related rehabilitation literature.

Type of evaluation	Definition	Example in literature
Cost-effectiveness analysis (CEA)	A comparison between the costs and an outcome for a specific treatment or intervention (e.g. the cost of providing a prosthesis or orthosis compared to not providing one)	Dobson, DaVanzo & Associates LLC: cost effectiveness of prosthesis among Medicare beneficiaries^17^
Cost utility analysis (CUA)	Often referred to as a sub-type of CEA; specifically, an analysis that includes health utility (i.e. health related quality of life or quality adjusted life year/QALY)	Gerzeli et al: Cost utility analysis comparing different microprocessor knees among working-age patients in Italy^18^
Cost benefit analysis (CBA)	An alternative to CEA, monetary value is placed on both costs of treatment and effectiveness; all costs (i.e. direct and indirect) are considered	Glassman et al.: costs and benefits (outcomes) of several non-surgical treatments compared for adult scoliosis^19^
Cost minimization analysis	An analysis conducted to identify a least costly alternative of effective treatment (e.g. tele-rehabilitation versus face to face)	^*^unable to identify a specific O&P example
Cost-consequence	An analysis used to describe an intervention or compare two or more interventions including the effect of costs and outcomes	Gil et al: Cost comparison of limb salvage versus amputation^20^ and Edwards et al.: Markov model assessing cost consequence of prosthetic rehabilitation^21^
Multi-criteria decision analysis or discrete choice experiment (preference studies)	A structured process for making decisions and is a tool that can extend traditional economic analysis methods to include the patient perspective and assist with prioritization of healthcare interventions	Geidl et al: Assessing exercise preferences for patients after a stroke^22^

Previous work has demonstrated that it is possible to assess healthcare resource utilization and costs through the use of a population-based, nationally validated claims dataset while providing meaningful insight into clinical care and patient outcomes.^17,23^ Furthermore, decision-making based on the preferences of patients (e.g. health state preference, utility) along with traditional economic analyses (e.g. cost-effectiveness studies) will contribute to optimizing patient outcomes. These types of health economic concepts are important to understand as the O&P practitioner and key stakeholders are continuing to navigate the increasingly challenging demands of the healthcare system.

### Rehabilitation services

Currently, there is limited evidence in O&P rehabilitation regarding outcome factors related to delivery of care such as patient preferences, accessibility and timing of provision, economic impact and value of rehabilitation services for people with functional impairment or decreased mobility.^24^ Without adequate evidence on the performance and effectiveness of O&P rehabilitation treatment, scrutiny of services will continue by policymakers and payers, potentially resulting in reduced access to needed services. 2

Physical medicine and rehabilitation service is a broad category in healthcare targeting a wide population (children, adults, and older people) with a range of conditions impacting function and participation, including diverse interventions (rehabilitation medicine, orthopedic surgery, physical therapy, occupational therapy, prosthetics, orthotics, and assistive devices) and outcomes.^25^ The primary goal of physical rehabilitation services is to address individual needs towards the reduction of symptoms and to promote independence in daily activities or participation, 26 which includes predisposing (e.g. demographic characteristics such as amputation level), enabling (e.g. environmental, social, health insurance status), and need (e.g. modifiable health status such as comorbid health conditions) factors, which each contribute towards overall rehabilitation use.^2,27^

Clinical and policy decisions about appropriate and optimal rehabilitation interventions require evidence on resource allocation, costs and effectiveness.^25^ Rehabilitation services are often undervalued by health systems due to being under-funded, under-researched and under-provided in many contexts.^2,25^ Lack of evidence and knowledge on patient outcomes due to physical rehabilitation services result in reduced access to appropriate services, which includes access to assistive devices (e.g. orthoses and prostheses) and physical therapy.^28,29^

### Lack of understanding of value of prosthetic rehabilitation

The demand for value-based care in rehabilitation is growing while the concept of value is multidimensional and may be defined differently depending on the stakeholder (e.g. patient, payer, provider or society). The traditional economic definition of value is dependent on cost, quality and willingness to pay for a good or service.^12^ The components that comprise how we gauge the value of an intervention or healthcare service is based on perspective, whether societal or individual, as well as cultural perceptions.^12,17^ Therefore, it is important to assess health interventions and resultant outcomes (functional health or economic) within the context of a single country or region. The approach to place value and quantify treatment effects is more widely being applied with payers and policymakers asking for evidence.^8,17^ The field of physical rehabilitation has perhaps fallen behind other services in the amount of value-based evidence.^2,13^ O&P care, a niche subset within physical rehabilitation, has arguably fallen even further behind. It is critical that as a field, - we collaboratively work to gauge value based on key benefits (including clinician and patient reported measures) that demonstrate real-world effectiveness of the interventions, as O&P devices are unique.

For example, consider post-amputation recovery, aside from differences in patient acuity, a high post-operative mortality rate suggests that quality improvement programs need to address the prosthetic rehabilitation needs. For instance, being mobile and physically active improves cardiovascular health, reduces the negative effects of diabetes and reduces depression or feelings of isolation.^30^ Patient satisfaction and quality of life are associated with less time between amputation surgery and delivery of a prosthesis.^31^ Furthermore, satisfaction and quality of life are correlated with mobility and patients with no prosthesis are unable to be as physically mobile.^31^ Without prosthetic care individuals have increased risk of clinical complications including increases in healthcare utilization and spending.^17^ Based on the current research, it is reasonable to propose that lack of prosthetic rehabilitation negatively influences mobility, satisfaction and quality of life. Further investigation is needed to establish why wearing a prosthetic device improves survival and potentially reduces overall utilization or economic burden.^32^ Without this data, there is an underappreciation for the true value of prosthetic rehabilitation.

### Standards of care and rehabilitation guidelines post-amputation

The standards of care post-amputation are limited aside from the immediate surgical care protocols. Furthermore, of the limited guidelines published, there is low physician adherence or awareness of the processes.^33^ HEOR studies can inform how a reduction in access to rehabilitation services reduces individual health outcomes. The integration of health economic studies and evidence into clinical practice guidelines adds a dimension that informs stakeholders (including patients) on how an intervention impacts costs, health outcomes and provides a way to evaluate potential consequences of practice. For example, there is no standard or regulated time from amputation surgery for when a lower limb prosthetic device should be provided or intervention initiated, such as a consult with a prosthetist.^29^ However, a recent study analyzed the impact of providing a prosthesis earlier, within 0 to 3 months post-amputation and demonstrated an overall cost savings.^23^ Additionally, there is not a standard guideline to what type of device is appropriate based on patient presentation.^5,33,34^ Future studies should compare selection and design of devices and include outcomes such as health utility and health-related quality of life. HEOR studies have the potential to inform clinical practice guidelines with the intention to optimize patient care and outcomes.

The recently published Mobility Analysis of AmpuTees (MAAT II) aims to assist in clinical decision-making by presenting standard outcome measures of mobility and demonstrates that the presence of comorbidities does not preclude an individual from prosthetic mobility success.^35^ Specific outcome measures are not standard of practice yet; however, the MAAT II study is a start to standardize prosthetic decision-making by demonstrating that the incorporation of patient outcomes is critical to inform policy.

The provision and use of a prosthesis is a critical component of a person's rehabilitation after a LLA as it is associated with a person's ability to return to ADLs and reintegrate into social or work routines.^17,36^ The timing from amputation surgery to initial device provision has several potential influences including the patient's age, income and rehabilitation setting.^36^ Post-acute care typically occurs at home, an in-patient rehabilitation facility or skilled nursing facility, all which contribute to varying processes and therefore influence timing.^36^

***Gaps in our knowledge***

In spite of the growing number of potential prosthesis users, the increasing number of individuals with functional impairment, and of those who experience fall-related injuries in the US, research in HEOR among O&P is sparse. Nationally, we lack the outcomes research, cost analyses and clinical practice guidelines needed to minimize acute health complications or emergency utilization, support patients' functional mobility, and reduce costs associated with less-than-optimal patient outcomes. Yet, the influence of O&P interventions on modifiable clinical outcomes, such as functional mobility or pain, are not well understood. There is a shortage of empirical outcomes research to demonstrate effectiveness and value of rehabilitation services for individuals with LLA.

### Informing Perspective on the Field

With increasing pressure from payers for clinicians to efficiently and effectively provide O&P care, continued RWE to demonstrate and describe the value of O&P rehabilitation is crucial.^17,24^ As O&P technology continues to improve and provide benefit to all O&P device users, manufacturers should work collaboratively with key stakeholders and clinical sites to sponsor and disseminate RWE studies. Such RWE studies would enhance findings from controlled clinical trials that are unable to capture the more broad-lived experiences of the naturally heterogeneous, diverse population of individuals with different O&P rehabilitation needs.

RWE studies should include elements that focus on the enabling factors and perceived needs to further inform on how devices are accessed, utilized, and the subsequent associated outcomes in diverse populations. RWE is a more useful tool to engage physicians and patients following specific product launches.^13^ Publishing observational studies of real-world data offers an important opportunity for researchers to provide stakeholders with data that reflects effectiveness in addition to existing evidence on efficacy and safety, particularly related to long-term outcomes. These types of studies can also include outcomes, such as patient-reported information and economic evidence (e.g. cost or utility data), which are growing in demand by payers and regulatory agencies.

## CONCLUSION

It is recommended that future studies consider the incorporation of data on additional enabling factors (e.g., income and education levels, social networks), perceived need factors (e.g., adherence to medication, use of prosthesis), and other modifiable risk factors (e.g., smoking). Social determinants continue to influence the health outcomes of adults who require O&P care. Illness that leads to amputation disproportionately affects persons with lower socioeconomic status, older patients, and persons who are racial/ethnic minorities, which results in disproportionately lower mobility and quality of life outcomes.^37^

As the demands increase in healthcare for value-based outcomes and RWE, it is imperative we continue to evaluate the impact of O&P rehabilitation services based on predisposing factors, enabling factors, and perceived need factors together. Determining the value of O&P rehabilitation will help patients improve access to appropriate, high quality, and beneficial prosthetic componentry in a timely manner. If O&P services result in cost avoidance, better clinical outcomes, and improved quality of life for patients with LLA or after a stroke, then we should continue to connect clinicians and researchers to inform administrative decision-making, improve coverage of services so all patients have equity in access and health outcomes.

## CALL TO ACTION

There is a growing number of prosthesis and orthosis users, individuals with functional impairment, and those who experience fall-related injuries in the US. Yet, research in HEOR among O&P struggles to keep pace. Nationally, there is a need for more outcomes research, cost analyses and clinical practice guidelines to help minimize acute health complications or emergency utilization, support patients' functional mobility, and reduce costs associated with less-than-optimal patient outcomes. The influence of O&P interventions on modifiable clinical outcomes, such as functional mobility or pain, needs a greater level of understanding. There is a need for more empirical outcomes research to demonstrate the effectiveness and value of rehabilitation services for individuals with functional impairment who require O&P devices.

The first call to action recommended is for clinical researchers and health outcomes researchers (e.g. epidemiologists, economists) to join together to assess effectiveness of O&P devices on diverse populations. A greater understanding of effectiveness and RWE will improve access for patients to appropriate technology. A second call to action is for our professional bodies along with clinicians, patients and advocates to expand awareness of outcomes research. This includes the analysis of administrative databases, clinical databases, electronic health records, and more by researchers. Funding for this type of research will be critical for our outcomes and evidence to keep pace with other areas of healthcare in evolving need and value.

## DECLARATION OF CONFLICTING INTERESTS

The authors declare no conflict of interest.

## SOURCES OF SUPPORT

None.

## AUTHOR SCIENTIFIC BIOGRAPHY

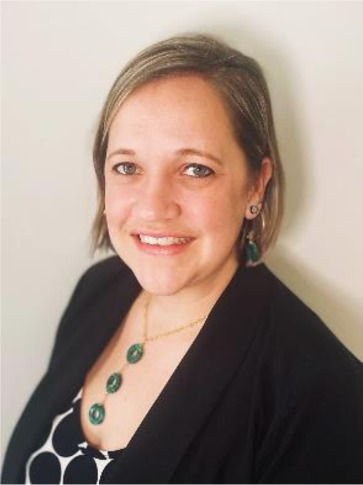



**Taavy Miller,** PhD, CPO, is a research scientist within Hanger's Department of Clinical and Scientific Affairs. Dr. Miller has broad experience working as a certified orthotist/prosthetist at large hospital-based systems and in private practice as well as teaching P&O at the university level. Dr. Miller holds a doctoral degree in Health Services Research with an emphasis in health economics and epidemiology. Her research focuses on health equity, reducing disparities and improving access through the assessment of health outcomes and effectiveness using administrative, clinical and patient reported data. She has published several studies in peer-reviewed journals and presented abstracts at national and international conferences.

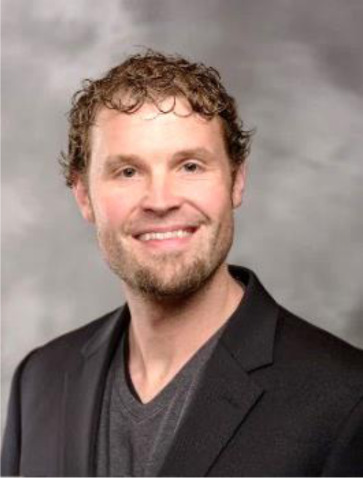



**Shane Wurdeman**, PhD, CP, is the Director of Clinical Research within Hanger's Department of Clinical and Scientific Affairs. He entered the field of O&P as a technician before transitioning to working as an orthotist/prosthetist and finally into his role as a principal investigator. Dr. Wurdeman holds a BS in physics, an MS in prosthetics and orthotics, and a PhD in biomechanics. He has co-authored more than 40 peer-reviewed manuscripts, published 3 book chapters, and presented more than 100 conference abstracts within the field of orthotic and prosthetic rehabilitation. He is a fellow with distinction of the American Academy of Orthotists and Prosthetists, from whom he was recognized in 2020 with their prestigious Academy Research Award. He currently serves as the Research Director for the American Orthotic and Prosthetic Association and Chair of the Center for Orthotic and Prosthetic Learning. He has been supported by private grants as well as government grants from the National Institutes of Health and Department of Defense.

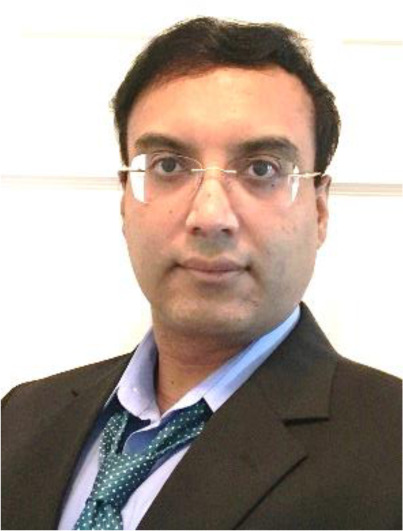



**Dr. Rajib Paul** is an associate professor of Biostatistics in the Department of Public Health Sciences and affiliate faculty of the School of Data Science at the University of North Carolina Charlotte. Dr. Paul has a broad spectrum of research interests. His areas of expertise include Bayesian methods, big data analysis, spatial and spatiotemporal statistics, stochastic computation (Markov Chain Monte Carlo algorithms), and the applications of statistics to environmental, epidemiological (public and community health), and health policy-related problems. After graduating from the Ohio State University with a Ph.D. degree in Statistics, he joined Western Michigan University (WMU) as a faculty in the Department of Statistics. He was an Associate Director and one of the founding members of the Health Data Research Analysis and Mapping (HDReAM) Center at the WMU. He has research experience in health disparities, environmental health, population health, and social and infectious disease epidemiology. His research focuses on identifying socioeconomic and geographic disparities in health outcomes and health service utilization. Dr. Paul worked on research projects funded by the National Science Foundation, Robert Wood Johnson Foundation, the Centers for Disease Control and Prevention, DHHS Health Resources and Services Administration (HRSA), and Blue Cross and Blue Shield of Michigan.

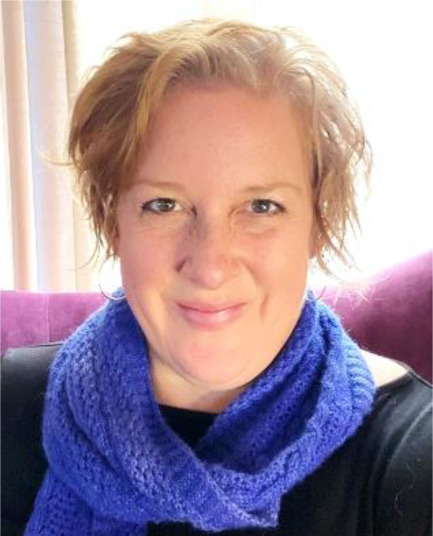



**Dr. Melinda Forthofer** is a Professor in the Department of Public Health Sciences in the College of Health and Human Services at the University of North Carolina Charlotte. From 2016–2020, Dr. Forthofer served as Department Chair, leading the department through period of exponential growth through the addition of new programs and concentrations and recruitment of new faculty. Prior to joining UNC Charlotte, Dr. Forthofer was on the faculty at the University of South Florida (1996-2006) and at the University of South Carolina (2006-2016). For over 20 years, her work has focused on social factors related to health behavior change in diverse community settings, often via community-based research. Much of her current research is focused on the role of social factors in the promotion of health behaviors, particularly the role of social networks in physical activity. Her research has been supported by over $29 million in extramurally funded research grants, via awards from several federal agencies (CDC, HRSA, NIH), state health departments, local and national nonprofit organizations, and several private sector organizations.
